# Functional promoter upstream p53 regulatory sequence of *IGFBP3 *that is silenced by tumor specific methylation

**DOI:** 10.1186/1471-2407-5-9

**Published:** 2005-01-20

**Authors:** Tadashi Hanafusa, Toshiyuki Shinji, Hidenori Shiraha, Kazuhiro Nouso, Yoshiaki Iwasaki, Eichiro Yumoto, Toshiro Ono, Norio Koide

**Affiliations:** 1 Okayama University Advanced Science Research Center, Department of Radiation Research, Shikata Laboratory, Shikata-cho 2-5-1, Okayama Japan; 2 Department of Laboratory Medicine, Okayama University Graduate School of Medicine and Dentistry. Shikata-cho 2-5-1, Okayama Japan; 3 First Department of Internal Medicine, Okayama University Graduate School of Medicine and Dentistry. Shikata-cho 2-5-1, Okayama Japan; 4 Department of Microbiology and Immunology, Albert Einstein College of Medicine, 1300 Morris Park Av, Bronx, NY 10461, USA

## Abstract

**Background:**

Insulin-like growth factor binding protein (IGFBP)-3 functions as a carrier of insulin-like growth factors (IGFs) in circulation and a mediator of the growth suppression signal in cells. There are two reported p53 regulatory regions in the *IGFBP3 *gene; one upstream of the promoter and one intronic. We previously reported a hot spot of promoter hypermethylation of IGFBP-3 in human hepatocellular carcinomas and derivative cell lines. As the hot spot locates at the putative upstream p53 consensus sequences, these p53 consensus sequences are really functional is a question to be answered.

**Methods:**

In this study, we examined the p53 consensus sequences upstream of the IGFBP-3 promoter for the p53 induced expression of IGFBP-3. Deletion, mutagenesis, and methylation constructs of IGFBP-3 promoter were assessed in the human hepatoblastoma cell line HepG2 for promoter activity.

**Results:**

Deletions and mutations of these sequences completely abolished the expression of IGFBP-3 in the presence of p53 overexpression. *In vitro *methylation of these p53 consensus sequences also suppressed IGFBP-3 expression. In contrast, the expression of IGFBP-3 was not affected in the absence of p53 overexpression. Further, we observed by electrophoresis mobility shift assay that p53 binding to the promoter region was diminished when methylated.

**Conclusion:**

From these observations, we conclude that four out of eleven p53 consensus sequences upstream of the IGFBP-3 promoter are essential for the p53 induced expression of IGFBP-3, and hypermethylation of these sequences selectively suppresses p53 induced IGFBP-3 expression in HepG2 cells.

## Background

Insulin-like growth factor binding protein (IGFBP)-3 is a multifunctional protein ferrying insulin-like growth factors (IGFs) in circulation and mediating growth suppression signals in cells. Serum IGFBP-3 protein (< 5000 ng/ml) complexes with IGFs and an acid labile subunit (ALS), to extend the half lives and modulate the bio-availability of IGFs [1]. While a precise mechanism of action is not clear, the growth suppressive activity of IGFBP-3 depends on its nuclear translocation [2]. Other growth suppressors such as p53, retinoic acids, transforming growth factor (TGF)-β, and tumor necrosis factor (TNF)-α induce IGFBP-3 as a mediator of growth suppression [3-6]. The growth suppression by IGFBP-3 is independent from the modulation of IGFs action [7-9]. The functional importance of the IGFBP-3 in the growth suppression is noteworthy.

IGFBP-3 is produced in most tissues, but the main site of production is liver. It is produced by non-parenchymal cells (endothelial and Kupffer cells) while parenchymal cells (hepatocytes) do not produce it under normal condition [10]. We postulate that IGFBP-3 is a gene induced by growth suppression signals such as p53 in hepatocytes. While the growth suppression imported by IGFBP-3 suggests the potential for tumor suppression, polymorphisms, but no significant mutations were observed in a survey of several tumors [11]. As gene silencing may occur without mutations, we recently investigated IGFBP-3 promoter hypermethylation in human hepatocellular carcinoma [12]. These promoter hypermethylations were subsequently reported in other tumors systems [13,14].

Promoter analysis of IGFBP-3 indicated that the NaB-RE sequence is essential for the sodium butyrate (NaB) induced IGFBP-3 expression [15], but the importance of the eleven upstream p53 binding sites reported by Bourdon *et al*. were not confirmed until now [16]. The methylation hot spot we identified exactly matched the putative p53 binding sites that Bourdon *et al*. indicated. Thus, we postulated that these sites are important for the expression of IGFBP-3 induced by p53. Moreover, we hypothesized that the suppression of apoptosis mediated by IGFBP-3 due to the promoter hypermethylation will be a possible pathway of hepatocarcinogenesis. To explore this possibility, the functions of the promoter upstream binding sites of p53 were examined precisely in this study.

## Methods

### Cell culture

HepG2 cells were obtained from Japanese Cancer Research Resources Bank (Tokyo, Japan), and maintained in D-MEM supplemented with 10 % FCS (Life Technologies, Tokyo, Japan), antibiotic-antimycotics at 37°C in a humidified atmosphere of 95 % air and 5 % CO_2_.

### Plasmids

pGL2-IGFBP-3, kindly provided by Dr. Youngman Oh (Oregon Health Sciences University, Portland, OR), carries a 1.9 kb IGFBP-3 promoter (-1805/+69) in pGL2-Basic (Promega Corp., Madison, WI). A series of deletion mutant constructs, pGL2-270, pGL2-240, pGL2-210, pGL2-180, pGL2-150, pGL2-120, pGL2-90, pGL2-60, pGL2-30, and pGL2-1 containing the indicated fragments upstream of the transcription start site and 60 bp of fragments downstream of the transcription start site, were generated by PCR amplification of the promoter fragment and subsequent subcloning of the *Mlu *I-*Bgl *II fragment to pGL2-Basic (Table 1, Fig. 2). The transcription start site of the IGFBP-3 promoter, +1, is based on the sequence determined by Cubbage *et al*. [17]. The plasmid containing site-directed mutations in putative p53 binding sites was generated by replacing the wild type *Mlu *I-*Xho *I fragment of pGL2-210 with a mutant fragment synthesized artificially (pGL2-210B, Table 1, Fig. 3). A fusion gene with site-directed methylation was constructed by methylating the *Mlu *I-*Xho *I fragment (-210/-174) of pGL2-210 *in vitro *with *Sss *I methylase (New England Biolabs, Inc., Beverly, MA), and reconstituting the fragment and unmethylated vector (Fig. 4). pCMV-p53 and pCMV-p53mt135 were obtained from BD-Biosciences (East Meadow Circle, Palo Alto, CA).

### Transient transfection

HepG2 cells were transiently transfected using the FuGENE 6 transfection reagent according to the manufacturer's instructions (Roche Molecular Biochemicals, Indianapolis IN). Cells were seeded at a density of 5 × 10^4 ^cells/well in 24-well plates. After 24 hours, cells were transfected with 0.25 μg/well of reporter plasmid DNA in serum-containing medium. Forty-eight hours post transfection, cells were washed twice with PBS and collected for luciferase assays. Transfections were performed in quadruplicate and experiments were performed at least two times.

### Luciferase assay

Luciferase activities of cell lysates were measured according to the manufacturer's instructions (Promega Corp. Madison, WI) using a liquid scintillation counter (Aloka, LSC-700, Tokyo, Japan). Luciferase activities were normalized for total protein determined using the Bradford Assay (Bio-Rad Laboratories, In., Hercules, CA).

### EMSA (electrophoresis mobility shift assay)

278 bp of the *Mlu *I-*Bgl *II fragment of pGL-210 were labelled using [α-^32^P]dCTP by end filling with Klenow fragment, and used for EMSA. Oligonucleotide DNAs (BP3WPSF: 5'-GGCTGCAGCG GGCGTGCGCA CGAGGAGCAG GTGCCCGGGC GAGTCTCGAG CTGCACGCCC CCGAGCTCGG-3', BP3WPSR: 5'-CCGAGCTCGG GGGCGTGCAG CTCGAGACTC GCCCGGGCAC CTGCTCCTCG TGCGCACGCC CGCTGCAGCC-3'), comprising the promoter sequence of -210/-149, were custom-made (Sigma-genosys Japan, Ishikari, Japan), annealed one hour at room temperature, and used as cold competitor in the assay. EMSA was performed in a 20 μl reaction containing 10 mM Tris (pH 7.5), 2.5% Glycerol, 50 mM KCl, 0.1 mM EDTA, 1 mM DTT, and in the presence of 50 ng/μl of double-stranded poly [d(I-C)], 5000 cpm (2 ng) of ^32^P-labeled probe DNA, and 2.5 μg of H_2_O_2 _treated MCF7 nuclear extract (Active motif LLC, Palomar, CA). The reaction mixture was incubated at 14°C for 20 min. 20 μl of each reaction mixture was then loaded onto a native 4 % polyacrylamide gel containing 0.5 × Tris-Glycine buffer (25 mM Tris, 190 mM Glycine, 1 mM EDTA pH 8.3), and electrophoresed at 14°C, 100 V for 1 hr. For the supershift assay, a p53 antibody (Ab-2, Oncogene Research Products, San Diego, CA) was used.

## Results

### Deletion analysis

**Figure 1:**
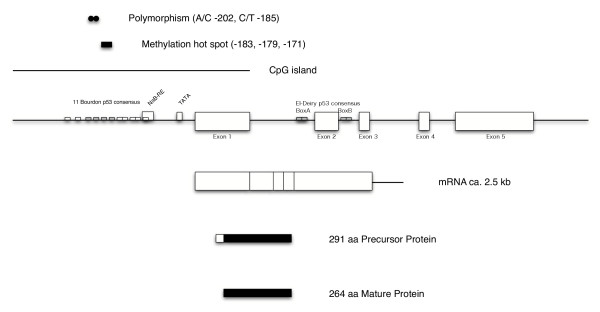
**Schematic genomic structure of the *IGFBP3 *gene**. There exist 11 p53 consensus sequences upstream of the promoter [16] and two binding sites in introns [3]. Polymorphisms and hypermethylations are identified in the promoter upstream p53 binding sites [26, 12].

**Figure 2:**
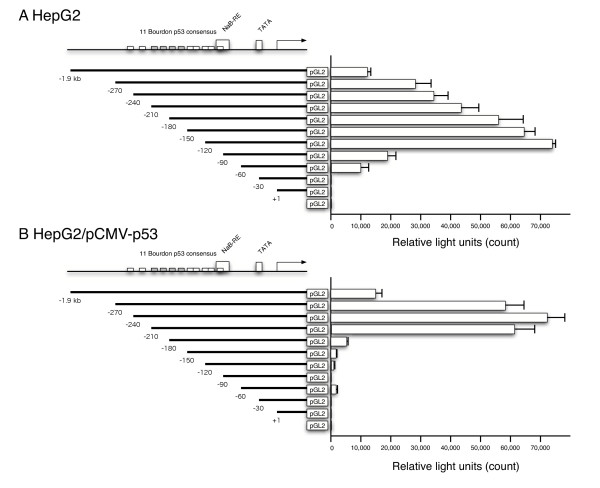
**Deletion analysis of IGFBP-3 promoter for the induction by p53**. HepG2 cells were transiently transfected with the panel of IGFBP-3 promoter-luciferase reporter constructs with (B) or without (A) co-transfection by pCMV-p53, and luciferase activity was measured after 48 h. Mean count of luciferase activity +/- SEM is shown.

We identified a hot spot of promoter hypermethylation in human hepatocellular carcinoma and its cell lines in the promoter upstream region of IGFBP-3 (Fig. 1). As methylation sites were identified to the cluster of putative p53 binding sites, we examined the role of these sites in IGFBP-3 expression. First, we constructed promoter deletion mutants of IGFBP-3 and examined the expression of the reporter gene in the absence (Fig. 2A) and presence (Fig. 2B) of p53 expression by the co-transfection of a p53 expression plasmid (pCMV-p53). As the transfection efficiency was not fully controlled, and p53 overexpression may to cause massive changes in cellular conditions, we did not compare results between the presence and absence of p53. Even though, we can obtain clear results about the effect of p53 to IGFBP-3 expression. The pattern of expression of deletion mutants was clearly different in the absence and presence of p53. In the absence of p53 overexpression, we identified the NaB-RE sequence as an essential site for expression, and deletion of p53 binding site enhanced the gene expression (Fig. 2A). These observations were similar to those of Walker *et al*. [15], but differed as HepG2 cell does not require NaB or Tricostatin A (TSA) for its activation. In contrast, in the presence of p53 over-expression, we identified that four p53 binding sites between -210 to -150 (relative transcription start site as +1) were essential for IGFBP-3 expression (Fig. 2B). When the deletion constructs were co-transfected with pCMV-p53mt135, the expression of the IGFBP-3 promoter was suppressed to a background level (almost same as blank constructs) in all constructs (data not shown). This indicates that IGFBP-3 expression we observe is tightly regulated by p53.

### Site-directed mutagenesis

**Figure 3:**
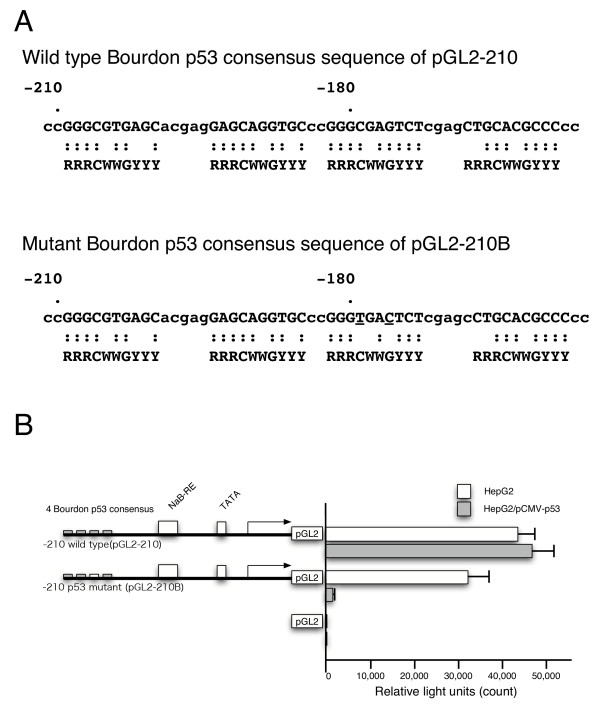
**Mutant analysis of IGFBP-3 promoter for the induction by p53**. A, Schematic of four wild type p53 consensus sequence of IGFBP-3 (-159/-209) of pGL2-210 and mutant sequence carrying point mutations in the core consensus sequence (-179 C to T, -176 G to C) of pGL2-210B. B, HepG2 cells were transiently transfected with wild type IGFBP-3 promoter-luciferase reporter constructs pGL2-210 or mutant IGFBP-3 promoter-luciferase reporter constructs pGL2-210B with (filled box) or without (open box) co-transfection by pCMV-p53, and luciferase activity was measured after 48 h. Mean count of luciferase activity +/- SEM is shown.

To confirm the importance of p53 binding sites between -210 to -150, we abrogated one of p53 binding sites by site-directed mutagenesis (C to T at -179 and G to C at -176, Fig. 3A). In the absence of p53 overexpression, there exist little differences in expression between the wild type and mutant construct (74 % relative to the wild type) (Fig. 3B). However, in the presence of p53 overexpression, IGFBP-3 expression was strongly decreased in the mutant construct (3.4 %) relative to wild type (Fig. 3B). For reasons already mentioned, we did not compare the result between in presence and absence of p53.

### Site-directed methylation

**Figure 4:**
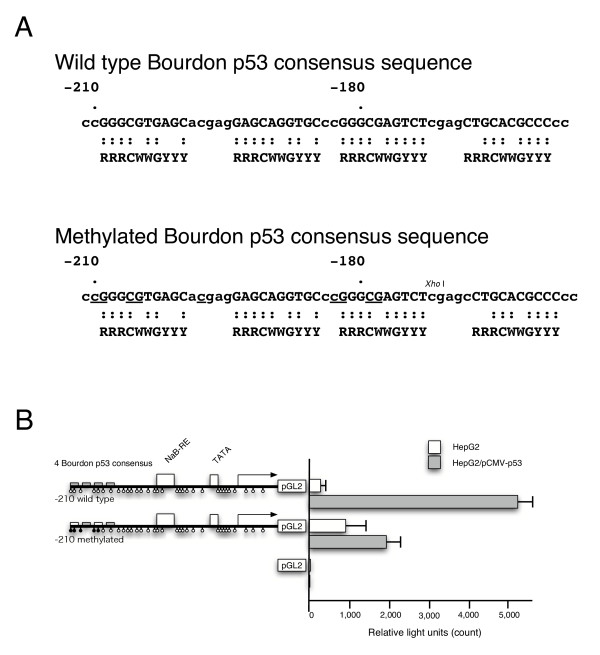
**Methylation analysis of IGFBP-3 promoter for induction by p53**. A, Schematic of four wild type p53 consensus sequence of IGFBP-3 (-159/-209) of pGL2-210 and a sequence of the methylated construct carrying the CpG methylation in the core consensus sequence (underlined) of pGL2-210. B, HepG2 cells were transiently transfected with wild type IGFBP-3 promoter-luciferase reporter construct or methylated IGFBP-3 promoter-luciferase reporter construct with (filled box) or without (open box) co-transfection by pCMV-p53, and luciferase activity was measured after 48 h. Mean count of luciferase activity +/- SEM is shown.

Next, we constructed *in vitro *methylated promoter constructs to evaluate the effect of methylation (Fig. 4A). For methylation, the *Mlu *I-*Xho *I fragment of pGL2-210 was methylated with *Sss *I methylase and reconstituted with unmethylated reporter vector fragment. As we used linear constructs for the transfection, the expression of luciferase was strongly suppressed compared to circular plasmids (0.7 %). But similar patterns compared to site directed mutation were observed. Although the expression of IGFBP-3 was slightly enhanced in the absence of p53 overexpression in the methylated construct, it was decreased in the presence of p53 overexpression in the methylated construct (Fig. 4B). In this experiment, at a glance, we observed induction of IGFBP-3 expression by p53, but the transfection efficiency of linear plasmids is low, while the relative levels of p53 and the availability of putative negative regulators are extremely different from other experiments. Thus, we cannot conclude in this case, whether or not there is induction by p53.

### EMSA

**Figure 5:**
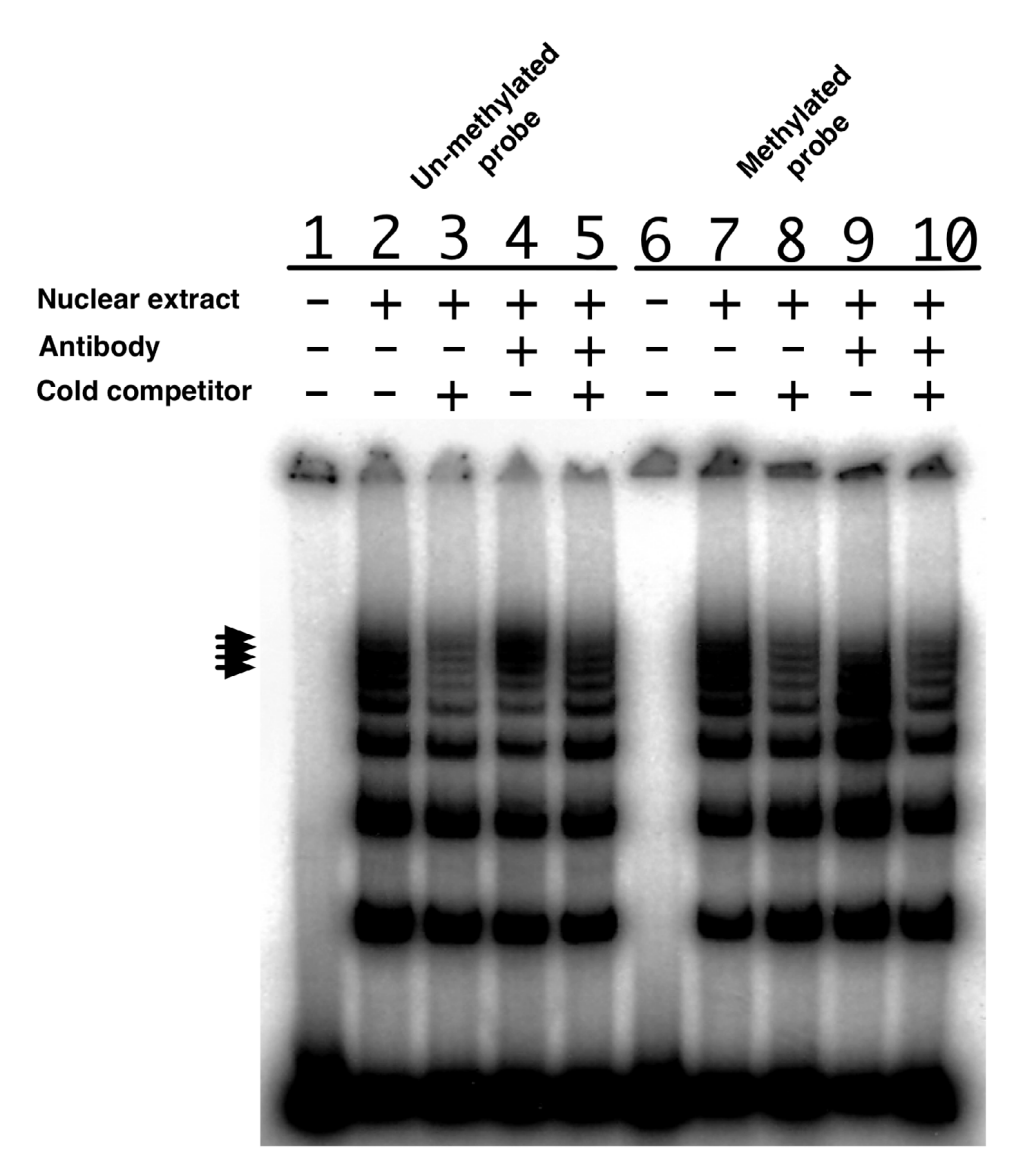
**Specific binding of p53 to IGFBP-3 promoter and its inhibition by methylation**. 2 ng of end-labelled 278 bp of *Mlu *I-*Bgl *II fragment of pGL-210 were used for EMSA. To construct the methylated probe, a labelled fragment was methylated *in vitro *with *Sss *I methylase. Each reaction contained the components listed in Materials and Methods. 2.5 μg of H_2_O_2_-treated MCF7 with an induced p53 expression was used as nuclear extract. Supershift was obtained using 0.1 μg of anti-p53 (Ab-2). As competitor, 200 ng of the fragment that carries the core p53 binding site (-210/-149) were used. The mixture was incubated at room temperature for 20 min and analyzed on 4 % nondenaturating polyacrylamide gel in 0.5 × Tris-Glycine buffer. Supershift bands observed in lane 4 were indicated by arrows.

EMSA was performed to determine whether p53 protein binding was disturbed by methylation. In this assay, the wild type and methylated promoter fragments (278 bp, -210/+60) were incubated with H_2_O_2_-treated MCF7 nuclear extract that express high level of p53. In the wild type promoter probe, we observed supershifted complex (Fig. 5, lane 4, indicated by arrows) when p53 antibody (Ab-2) is added to the reaction mixture. A 100-fold molar excess of a cold 70 bp sequence containing a p53 binding site (-210/-149), competed the probe (Fig. 5, lane 5). In methylated probe, we observed no supershifted complex (Fig. 5, lane 9). As we used probes that contain a Sp1/GC box and TATA box, we observed the shift and supershift bands in the methylated probe as well as unmethylated probe. These bands were postulated to be due to p53 binding to the nuclear factor such as p300. These observations indicate that p53 binding to the *IGFBP3 *promoter sequence is blocked or at least attenuated by hypermethylation.

## Discussion

**Figure 6:**
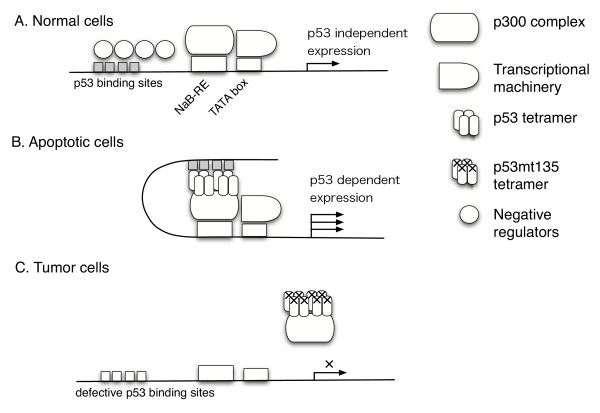
**Working model of p53 action to IGFBP-3 promoter**. In normal cells, IGFBP-3 is induced by such as growth hormone and IGFs, and steady level of expression is observed in some cells. As the deletion of p53 binding sites enhances the expression of IGFBP-3, the binding of negative regulators is expected (Fig. 6A). In apoptotic cells, p53 tetramers at high level of expression bind to upstream binding sites. These tetramers recruit the p300 complex to its binding site, and a p53 dependent high level of expression is observed (Fig. 6B). In tumor cells, deletions, mutations, methylations of p53 binding sites, or mutations of p53 such as p53mt135, disturb the binding of p53 tetramers to its binding sites, and this prevents the binding of p300. As a result, the expression of IGFBP-3 is suppressed in tumor cells.

We have indicated that the p53 binding sites upstream of IGFBP-3 promoter are essential for its induction by p53, and that the induction can be suppressed by promoter hypermethylation in the human hepatoblastoma cell line HepG2. A working model of the p53 action in IGFBP-3 promoter upstream binding sites is summarized in Fig. 6. In this model, in normal cells, IGFBP-3 is induced by factors such as growth hormone (GH) and IGFs, and steady levels of expression are observed in some cells. That the deletion of p53 binding sites enhances the expression of IGFBP-3, suggests existence of negative regulators (Fig. 6A). In apoptotic cells, p53 tetramers at high levels of expression bind to upstream binding sites. These tetramers recruit the p300 complex to its binding site, thereby a p53-dependent high level of expression (Fig. 6B). In tumor cells, deletions, mutations, methylations of p53 binding sites, or mutations of p53 such as p53mt135, disturb the binding of p53 tetramers to their binding sites, and this prevents the binding of the p300 complex to IGFBP-3 promoter. As a result, the expression of IGFBP-3 is suppressed in tumor cells (Fig.6C). In this model, promoter hypermethylation has the same effect as promoter mutation in determining IGFBP-3 expression.

Our observations of promoter hypermethylation in human hepatocellular carcinomas and derivative cell lines (12), and the observations in this report strongly support the notion that *IGFBP3 *is a true tumor suppressor gene. *IGFBP3 *is a gene that is silenced by biallelic hypermethylation or hypermethylation and loss of heterogeneity (LOH) in human hepatocellular carcinoma. As reported recently [14], we have also observed the reduced expression of IGFBP-3 in several tumors such as, breast (9/41), uterus (11/42), ovary (6/16), kidney (6/20), and prostate (1/4) using the cancer profiling array (BD bioscience, data not shown). We therefore postulate that the tumor suppressor role of IGFBP-3 will not be limited to HCCs. In addition, there are also many reports of IGFBP-3 overexpression in tumors from breast [18], prostate [19], kidneys [20], and lung squamous cells [21], so on. We thus anticipate the existence of additional defects, such as papilloma virus infections that inactivate IGFBP-3 [22], TGF-β / Rb signalling abnormalities that often coincide with IGFBP-3 overexpression [23-25], or as yet unknown defects in IGFBP-3 receptor function leading to IGFBP-3, for these overexpression in tumors.

IGFBP-3 is a ubiquitous, multifunctional protein, whose importance as a carrier of IGFs is evident. The absence of gross loss-of-function mutations of IGFBP-3 observed to date likely underscores its functional importance. We hypothesize that IGFBP-3 is a gene whose basal level of expression is essential for cell survival, but upon induction by p53, high levels of expression of IGFBP-3 induces apoptosis. Alternatively, IGFBP-3 may be a gene that is essential for cell survival when induced by growth hormones or IGFs, but functions as an apoptotic mediator when induced by p53.

We observed slight base changes within p53 binding sites strongly influenced the induction of IGFBP-3 by p53. As SNPs that change the expression level of IGFBP-3 were within p53 binding sites [26], and it was reported that the IGFBP-3 is differentially activated by p53 mutants [27,28], we postulate that the expression and functions of IGFBP-3 is controlled in some way by p53 binding sites in the promoter of IGFBP-3. This may include the intronic p53 binding sites as well as the upstream sites explored here. IGFBP-3 may, therefore, have an important function in tumor development through p53 control.

We identified the MyoD (-195/-186) and WT1 (-164/-156) binding sites as well as p53 binding sites at the hypermethylation hot spot in HCC by promoter analysis using TRANSFAC (v 4.0). As MyoD is a transcription factor that can induce apoptosis, and WT1 is a tumor suppressor gene, the existence of a binding site for these putative regulator genes in the hot spot of the promoter of IGFBP-3 suggest the possibility that these genes also use IGFBP-3 as a mediator of their actions.

## Conclusions

We conclude that four out of eleven p53 consensus sequences upstream of the IGFBP-3 promoter are essential for the p53 induced expression of IGFBP-3, and hypermethylation of these sequences selectively suppresses p53 induced IGFBP-3 expression in HepG2 cells. As IGFBP-3 functions downstream of many growth suppressors and its growth suppression effects are drastic, and as it is a small-sized secreted protein, the use of IGFBP-3 in tumor therapy will be a promising option.

## Abbreviations

ALS; acid labile subunit, D-MEM; Dulbecco's modification of Eagle's medium DTT; dithiothreitol, EDTA; ethylenediaminetetra-acetic acid, EMSA; electrophoresis mobility shift assay, FCS; fetal calf serum, HCC; hepatocellular carcinoma, IGF; Insulin-like growth factor, IGFBP-3; Insulin-like growth factor binding protein-3, LOH; loss of heterogeneity, NaB; sodium butyrate, NaB-RE (sodium butyrate-responsive region), PBS; phosphate buffered saline, PCR; polymerase chain reaction, TGF-β; transforming growth factor-β, TNF-α; tumor necrosis factor-α, SEM; standard error of mean, SNP; single nucleotide polymorphism.

## Competing interests

The author(s) declare that they have no competing interests.

## Authors' contributions

TH carried out these studies and manuscript preparation, KN and YI participated plasmid construction and reporter assay, TS and HS participated the EMSA, EY helped the array study, TO participated in the design of the study and performed the statistical analysis. NK conceived of the study, and participated in its design and coordination and helped to draft the manuscript. All authors read and approved the final manuscript.

## Pre-publication history

The pre-publication history for this paper can be accessed here:



## Figures and Tables

**Table 1:** Oligonucleotide DNAs used for construction of IGFBP-3 promoter mutants.

**Plasmid**	Cloned fragment*	sense primer** (5' to 3')	antisense primer** (5' to 3')
pGL2-270	-270/+60	ggacgcgtctggagtgccggggtggc	ggagatctgctgtggaatccaggcag
pGL2-240	-240/+60	ggacgcgtggttcttgtagacgacaa	ggagatctgctgtggaatccaggcag
pGL2-210	-210/+60	ggacgcgtcgggcgtgagcacgagga	ggagatctgctgtggaatccaggcag
pGL2-180	-180/+60	ggacgcgtgcgagtctcgagctgcac	ggagatctgctgtggaatccaggcag
pGL2-150	-150/+60	ggacgcgtggccccggctgctcaggg	ggagatctgctgtggaatccaggcag
pGL2-120	-120/+60	ggacgcgtcccgcagccgtgcctgcg	ggagatctgctgtggaatccaggcag
pGL2-90	-90/+60	ggacgcgtcctcccaacccccactcc	ggagatctgctgtggaatccaggcag
pGL2-60	-60/+60	ggacgcgtccggggcgtgtcctgggc	ggagatctgctgtggaatccaggcag
pGL2-30	-30/+60	ggacgcgttatatacgggccggcgcg	ggagatctgctgtggaatccaggcag
pGL2-1	+1/+60	ggacgcgtagatgcgagcactgcggc	ggagatctgctgtggaatccaggcag
pGL2-210B***	-210/+60	ggacgcgtctgcagcgggcgtgagcacga ggagcaggtgcccgggtgactctcgagct	agctcgagagtcacccgggcacctgctcc tcgtgctcacgcccgctgcagacgcgtcc

*; The number is indicated by transcription start site as +1.

**; *Mlu *I restriction site is included in sense primers and *Bgl *II restriction site is included in antisense primers.

***; pGL-210B was made by replacing *Mlu *I-*Xho *I fragment of pGL2-210 with the oligonucleotide DNA indicated.
